# Live imaging of follicle stimulating hormone receptors in gonads and bones using near infrared II fluorophore†
†Electronic supplementary information (ESI) available. See DOI: 10.1039/c6sc04897h



**DOI:** 10.1039/c6sc04897h

**Published:** 2017-03-06

**Authors:** Yi Feng, Shoujun Zhu, Alexander L. Antaris, Hao Chen, Yuling Xiao, Xiaowei Lu, Linlin Jiang, Shuo Diao, Kuai Yu, Yan Wang, Sonia Herraiz, Jingying Yue, Xuechuan Hong, Guosong Hong, Zhen Cheng, Hongjie Dai, Aaron J. Hsueh

**Affiliations:** a Program of Reproductive and Stem Cell Biology , Department of Obstetrics and Gynecology , Stanford University School of Medicine , Stanford , CA 94305 , USA . Email: aaron.hsueh@stanford.edu; b Department of Chemistry , Stanford University , Stanford , CA 94305 , USA . Email: hdai@stanford.edu; c Key Laboratory of Virology , Hubei Province Engineering and Technology Research Center for Fluorinated Pharmaceuticals , Wuhan University School of Pharmaceutical Sciences , Wuhan 430071 , China; d Molecular Imaging Program at Stanford (MIPS) , Bio-X Program , Department of Radiology , Stanford University , Stanford , CA 94305 , USA . Email: zcheng@stanford.edu

## Abstract

*In vivo* imaging of hormone receptors provides the opportunity to visualize target tissues under hormonal control in live animals. Detecting longer-wavelength photons in the second near-infrared window (NIR-II, 1000–1700 nm) region affords reduced photon scattering in tissues accompanied by lower autofluorescence, leading to higher spatial resolution at up to centimeter tissue penetration depths. Here, we report the conjugation of a small molecular NIR-II fluorophore CH1055 to a follicle stimulating hormone (FSH-CH) for imaging ovaries and testes in live mice. After exposure to FSH-CH, specific NIR-II signals were found in cultured ovarian granulosa cells containing FSH receptors. Injection of FSH-CH allowed live imaging of ovarian follicles and testicular seminiferous tubules in female and male adult mice, respectively. Using prepubertal mice, NIR-II signals were detected in ovaries containing only preantral follicles. Resolving earlier controversies regarding the expression of FSH receptors in cultured osteoclasts, we detected for the first time specific FSH receptor signals in bones *in vivo*. The present imaging of FSH receptors in live animals using a ligand-conjugated NIR-II fluorophore with low cell toxicity and rapid clearance allows the development of non-invasive molecular imaging of diverse hormonal target cells *in vivo*.

## Abstract 2

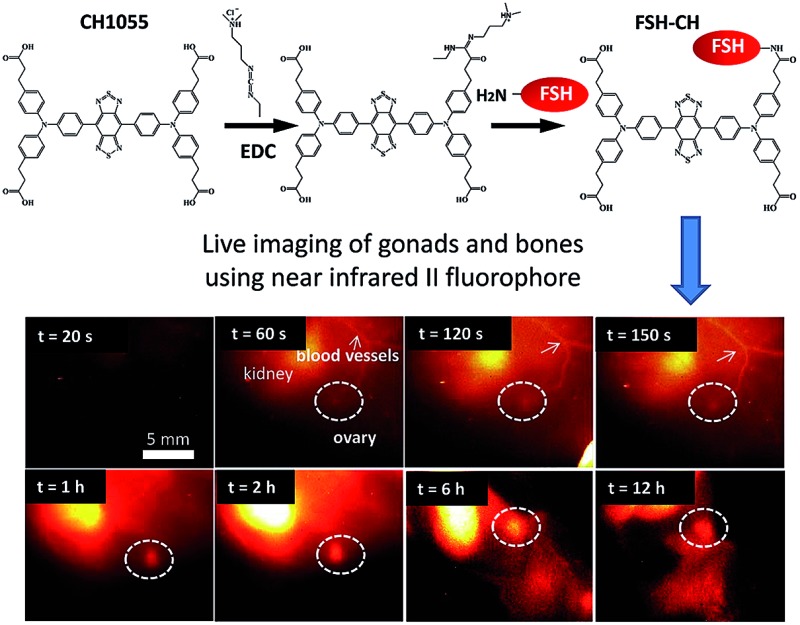
 *In vivo* imaging of hormone receptors provides the opportunity to visualize target tissues under hormonal control in live animals.

## 

Diverse peptide hormones act on their specific receptors in target cells to regulate different physiological functions. In addition to mediating hormonal actions, target cell receptors provide unique markers for imaging and identification of unique cell types in normal and malignant tissues. The follicle stimulating hormone (FSH) is a heterodimeric glycoprotein essential for gonadal development as it binds to specific receptors in granulosa cells of ovarian follicles and Sertoli cells of testicular seminiferous tubules.^1–3^ Ovaries contain follicles as functional units. Once activated to grow, follicles develop through primordial, primary and secondary (preantral) stages until they acquire an antral cavity, followed by further growth into preovulatory follicles capable of releasing a mature egg for fertilization.^4^ Imaging of ovarian follicles in patients has relied upon transvaginal ultrasound but the approach is limited in spatial resolution and only detects antral and larger follicles.^5^ For infertile female patients, it is important to assess the presence of preantral follicles because recent studies have indicated the possibility to promote preantral follicle growth for infertility treatment.^6,7^ In the testes, Sertoli cells are essential for spermatogenesis^8^ and monitoring of FSH receptors in these cells could allow for better diagnosis of male infertility.

In addition to FSH receptors in gonads, low levels of FSH receptors were found in cultured osteoclasts and these receptors have been proposed to promote osteoporosis in postmenopausal women.^9^ However, these findings were challenged due to an inability to confirm the low expression of FSH receptors in osteoclasts^10,11^ and difficulties occurred in performing *in vitro* FSH binding of the rigid bone structures. In view of the major health care costs incurred worldwide in both aging female and male populations,^12^ it is important to further elucidate the function of FSH receptors in bones.

Recent advances in biological imaging using novel fluorescent agents in the long NIR-II region have achieved a reduction of photon scattering and auto-fluorescence in tissues, thus reaching deeper penetration depths *in vivo*. Fluorescence imaging of biological systems in the NIR-II window can probe centimeter tissue depths and achieve micron-scale spatial resolution at millimeter depths.^13,14^ Using this approach, through-scalp and through-skull fluorescence imaging of mouse cerebral vasculature allowed real-time assessment of blood flow anomalies in a mouse cerebral artery occlusion stroke model.^15^

Here, we developed a ligand-based imaging dye by covalently linking a NIR-II fluorophore to FSH for *in vivo* imaging of FSH receptors in live animals. In addition to detecting ovarian follicles of different sizes and testicular seminiferous tubules, we conclusively demonstrated the expression of FSH receptors in vertebrae and other bone structures.

Recombinant human FSH (35.5 kDa) was conjugated to NIR-II dye CH1055 (M.W. 0.97 kDa) based on the 1-ethyl-3-(3-dimethylaminopropyl)carbodiimide hydrochloride (EDC) method^16^ (Fig. 1A). The UV-vis-NIR absorption spectrum of the FSH-CH solution in water exhibited an absorption peak at *ca.* 700 nm, while the fluorescence emission spectrum showed a main emission peak at 1055 nm, displaying a large Stokes shift of ∼400 nm (Fig. 1B). FSH is responsible for follicle growth^17^ and FSH receptors are expressed mainly in granulosa cells of ovarian follicles and Sertoli cells of testes tubules.^18^ We obtained granulosa cells by puncturing antral follicles of immature mice pretreated with equine gonadotropin (eCG) for 2 days to stimulate follicle growth. Following the centrifugation and sonication of cell suspensions, we prepared lysates from granulosa cells containing FSH receptors. Lysates of granulosa cells were printed on plasmonic, NIR fluorescence-enhancing Au slides^19–21^ to form reverse phase microarrays before incubation with FSH-CH. As shown in Fig. 1C, granulosa cell lysates displayed bright specific NIR-II signals as compared with the negligible signals from the lysates of U87MG glioblastoma cells. Also, cultured granulosa and MDA-MB-468 (MDA) mammary cancer cells were incubated with 500 nM FSH-CH at room temperature for 4 h followed by washing out of unbound ligands. As shown in Fig. 1D, strong signals were found in granulosa cells but not in MDA cells. Furthermore, the addition of a 10-fold excess of non-conjugated FSH together with FSH-CH blocked NIR-II signals (Fig. 1E and F), demonstrating the ability of non-conjugated FSH to compete for ligand binding.

**Fig. 1:**
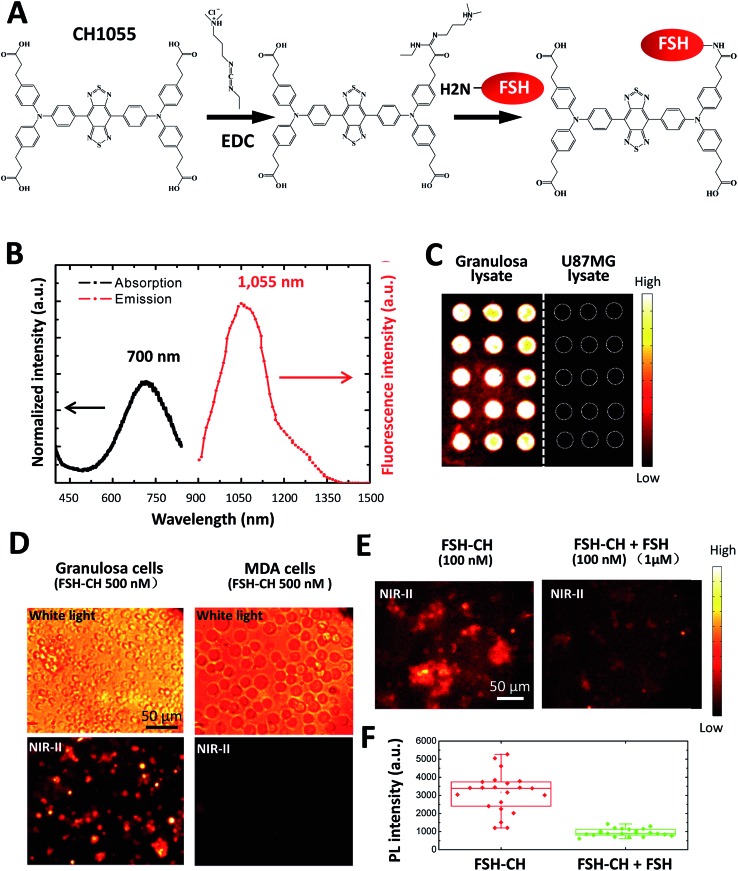
Conjugation of the follicle stimulating hormone (FSH) to the NIR-II CH1055 fluorophore (CH), and binding of the conjugates to FSH receptors *in vitro*. (A) Conjugation of FSH to CH1055 to derive FSH-CH using EDC, 1-ethyl-3-(3-dimethylaminopropyl)carbodiimide hydrochloride. (B) Absorption and emission spectra of FSH-CH, featuring a large Stokes shift of ∼400 nm. (C) Binding of FSH-CH to lysates of ovarian granulosa, but not to human glioblastoma U87MG cells, printed on plates. Granulosa cells were obtained from puncturing the ovaries of immature mice treated with equine gonadotropin (eCG) for 48 h. (D) Binding of FSH-CH to cultured ovarian granulosa, but not to MDA-MB-468 mammary cancer (MDA) cells. Upper panel shows bright field views whereas the lower panel shows NIR-II signals. Cells were cultured for 2 h at 37 °C before incubation with 500 nM FSH-CH. Unbound dyes were removed by washing cells 3 times using PBS. (E) Competition of FSH-CH binding to cultured granulosa cells by a 10-fold excess of non-conjugated FSH. (F) Quantitation of the NIR-II fluorescence intensity of FSH-CH binding to cultured granulosa cells by a 10-fold excess of non-conjugated FSH. Errors bars indicate the standard deviation of each group.

As shown in Fig. 2A and Video S1,† the injection of FSH-CH into adult female mice led to an initial accumulation of signals (at 60 seconds) in the kidney, the site of FSH metabolism.^22^ At 120 seconds after injection, signals appeared in the ovary, reaching their highest levels at 6 h and lasting for 24 h (Fig. 2B). Under high magnification *in vivo*, strong signals could be detected in ovarian follicles (Fig. 2C, *in vivo*) as confirmed by removing the ovary outside the body (Fig. 2C, *ex vivo*). Under confocal fluorescence NIR-II imaging using a home-built instrument, strong signals were found in granulosa cells of antral and smaller follicles (Fig. 2D, arrowheads). Furthermore, injection of FSH-CH together with a 20-fold excess of non-conjugated FSH led to lower signals in the ovary either *in vivo* or after its removal from the body (*ex vivo*) (Fig. 2E). Quantitation of the NIR-II fluorescence intensity indicated a major suppression of signals after blocking with excess non-conjugated FSH (Fig. 2F), demonstrating the hormonal specificity of FSH-CH binding.

**Fig. 2:**
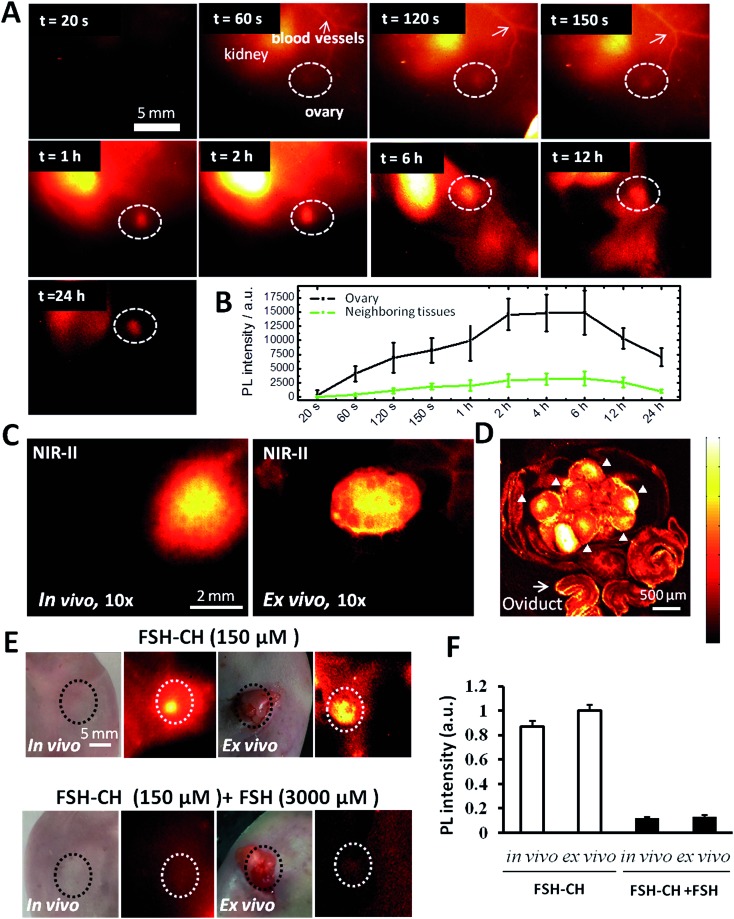
NIR-II imaging of ovarian follicles using follicle stimulating hormone-fluorophore CH1055 (FSH-CH) in adult female mice: time-course and specificity. (A) FSH-CH (12.5 μg) was injected into the tail vein of an adult female mouse before NIR-II imaging at different post-injection times. There was a rapid accumulation of signals in the blood vessels and kidney at 60 s. Ovarian signals begin to show up at 120 s and peak at 2–6 h after injection, showing a sustained retention for up to 24 h. Side view images are shown to focus on one ovary. (B) Quantitation of the fluorescence intensity in the ovary at different time points (*n* = 3). (C) Imaging at high magnification at 24 h after FSH-CH injection. Ovarian NIR-II signals inside the body (*in vivo*) and after ovary exposure from the abdominal cavity with the uterus, vasculature and nerves connected (*ex vivo*) are shown. (D) Confocal image of the same ovary showing bright signals in granulosa cells inside individual follicles (arrowheads) together with background signals in the oviduct (arrow). (E) Displacement of FSH-CH binding by non-conjugated FSH in the ovary. Adult female mice were injected with FSH-CH (12.5 μg of FSH) or FSH-CH plus a 20-fold excess of unconjugated FSH (250 μg) before imaging 2 h later. Strong NIR-II signals were found in the ovary both *in vivo* and *ex vivo* whereas no signal was found in the ovary when excess FSH was injected together with FSH-CH. Light microscope pictures accompany each NIR-II image. (F) Quantitation of NIR-II signals in individual groups. Error bars indicate the standard deviation of each group. PL, photoluminescence.

To further demonstrate the ability of FSH-CH to image preantral follicles, we injected FSH-CH into mice at 17 days of age, the ovaries of which contained only preantral or smaller follicles. As shown in Fig. 3A and B, strong signals were found in the ovary at both 2 h and 24 h after FSH-CH injection. Under confocal fluorescence imaging at high magnification, strong NIR-II signals were evident in secondary follicles (Fig. 3C and D, arrows).

**Fig. 3:**
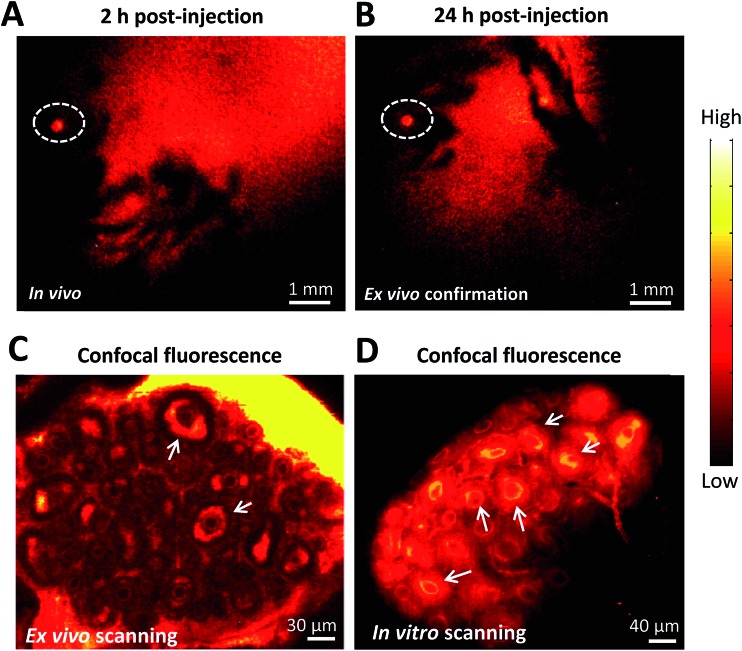
NIR-II imaging of ovarian follicles using follicle stimulating hormone-fluorophore CH1055 (FSH-CH) in immature female mice. A mouse at 17 days of age containing only secondary and smaller follicles in ovaries was injected with FSH-CH (6.25 μg) for 2 h (A) or 24 h (B) before NIR-II imaging. Confocal fluorescence microscope imaging under *ex vivo* ((C) ovary exposed from the abdominal cavity with the uterus, vasculature and nerves connected) and *in vitro* ((D) ovary removal from the body) conditions showed signals in the granulosa cells of secondary follicles (arrows).

To investigate FSH-CH binding to testes, adult male mice were injected with FSH-CH, followed by live imaging (Fig. 4A). Quantitation of NIR-II signals demonstrated accumulation of FSH-CH in both testes in a time-dependent manner with bright signals detected from 120 s onward, which peaked at 0.5 h and lasted for at least 2 h (Fig. 4B, dashed circles). Strong signals were confirmed after removing the testes from the body (Fig. 4C; *in vitro*). As shown in Fig. 4D, confocal fluorescence NIR-II imaging allowed *ex vivo* detection of seminiferous tubules. Furthermore, an injection of a 20-fold excess of FSH together with FSH-CH led to negligible signals in the testes (Fig. 4E), demonstrating the hormonal specificity of FSH-CH binding.

**Fig. 4:**
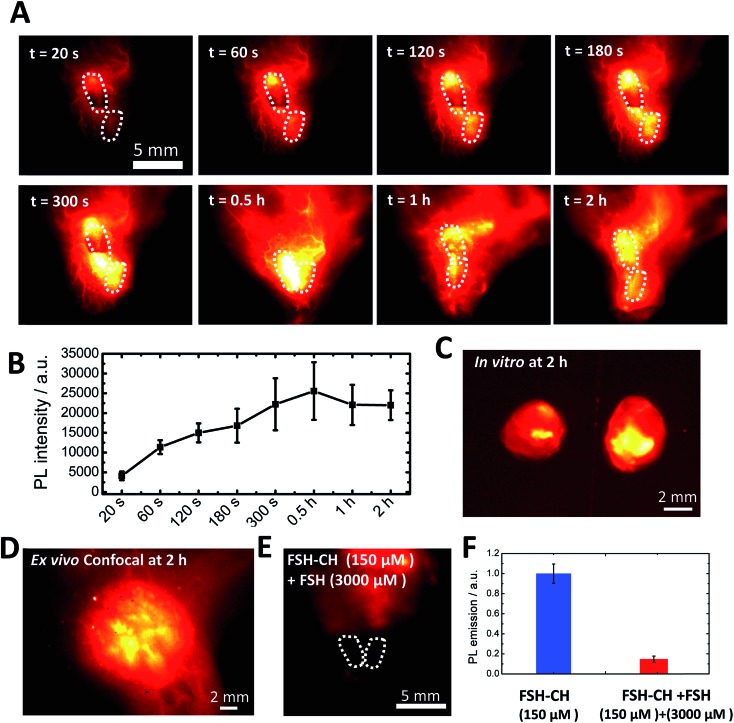
NIR-II imaging of testicular tubules using follicle stimulating hormone-fluorophore CH1055 (FSH-CH). (A) An adult male mouse was injected with 12.5 μg FSH-CH and imaged for up to 2 h showing strong signals in both testes (dashed circles). NIR-II signals began to show up at 60 s after tail injection and were maintained for up to 2 h. (B) Quantitation of the fluorescence intensity in the testes at different time points (*n* = 3). (C) *Ex vivo* images of the testes after removing them outside the body with vascular connection still attached. (D) Enlarged image showing confocal imaging of FSH-CH signals in testicular tubules. (E) Displacement of FSH-CH binding by FSH in the testes. Adult male mice were injected with FSH-CH or FSH-CH together with a 20-fold excess of non-conjugated FSH before imaging 2 h later. (F) Quantitation of NIR-II signals in individual groups. Error bars indicate the standard deviation of each group. PL, photoluminescence.

After injecting FSH-CH in both adult female and male mice, we found strong NIR-II signals in vertebrae and other bones. As shown in Fig. 5A, NIR-II signals were found in the spine, thighbones, tibia and ovaries in the female animals. In addition, nonspecific signals were found in the kidney and liver, the organs of FSH-CH metabolism. In the male animals, NIR-II signals were also found in the spine and thighbones (Fig. 5B). Under high magnification, NIR-II signals were detected in the vertebrae and thighbone of a female, together with the radius, ulna, and hand bones as well as the tibia and foot bones in a male (Fig. 5C). Furthermore, an injection of FSH-CH together with a 20-fold excess of FSH decreased the signals in the bones in both sexes (Fig. S2†), demonstrating the hormonal specificity of FSH-CH binding. Pharmacokinetics of bone (Fig. S3A†), based on NIR-II imaging using FSH-CH, has been shown. In addition, quantitation of the fluorescence intensity in the bone at each time point in both sexes is presented in Fig. S3D.†

**Fig. 5:**
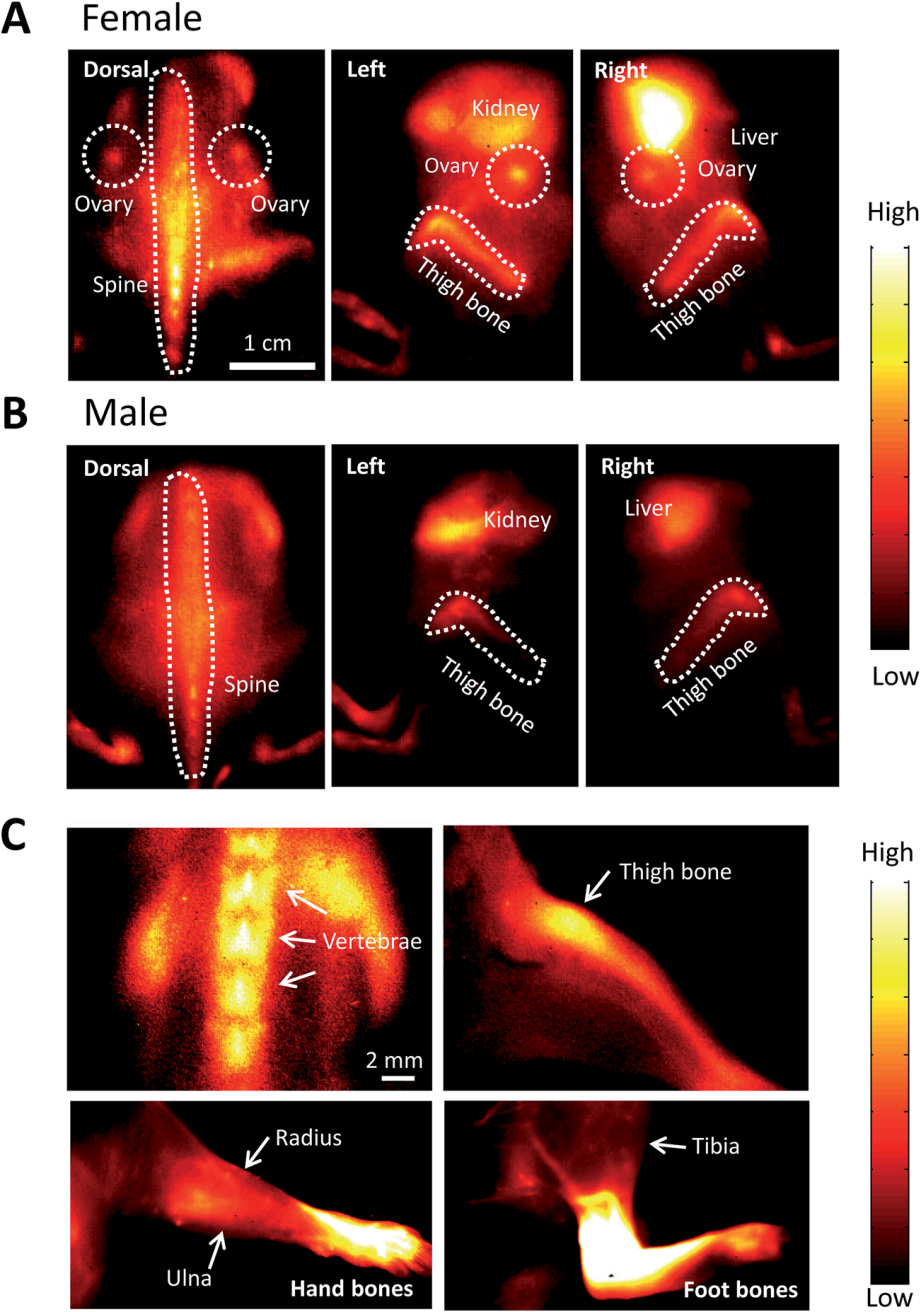
NIR-II imaging of bones using follicle stimulating hormone-fluorophore CH1055 (FSH-CH) in adult female and male mice. FSH-CH (12.5 μg) was injected into the tail vein of mice before imaging bones at 2 h post-injection. (A) For female mice, a dorsal view shows strong NIR-II signals in the ovaries and spine. In the left side view, NIR-II signals were found in the ovary and thighbone together with nonspecific signals in the kidney, the site of FSH metabolism. In the right side view, NIR-II signals were found in the ovary and thighbone together with strong signals in the liver, the site of CH1055 metabolism. (B) For male mice, a dorsal view shows NIR-II signals in the spine. The left side view shows NIR-II signals in the thighbone together with non-specific signals in the kidney. The right side view shows NIR-II signals in the thighbone together with nonspecific signals in the liver. (C) High magnification of the vertebrae and thighbone of a female, together with the radius, ulna, and hand bones as well as the tibia and foot bones in a male.

The maximum fluorescence signal strength of a structure imaged using a ligand-conjugated fluorophore is dictated by parameters including the ligand-receptor binding kinetics, the permeation of the imaging agent into a given tissue, imaging depth, and background autofluorescence of the imaging agent’s optical properties. In addition to the use of a NIR-II fluorophore that confers improvements in the spatial resolution due to the reduction in scattering and better penetration of signals at longer wavelengths, the present use of a FSH-conjugated NIR-II fluorophore allows high affinity and specific targeting of the fluorophore to unique cell types in gonads and bones expressing FSH receptors. The small size of the fluorophore does not interfere with the high affinity binding of FSH to its cognate receptors nor impedes FSH permeation to target cells. FSH receptors are expressed mainly in ovarian granulosa cells in females and Sertoli cells in testicular seminiferous tubules in males. In addition to providing the first *in vivo* imaging of FSH receptors in gonads, we further demonstrated FSH binding signals in vertebrae and other bones, consistent with earlier findings in isolated osteoclasts.^9^ In addition, NIR-II signals for FSH-CH were concentrated in the kidney and liver, the sites of FSH and CH1055 metabolism, respectively.^22^

Although antral preovulatory follicles could be stimulated by gonadotropins to release mature oocytes capable of undergoing fertilization for pregnancy, recent studies indicate that many infertile patients still possess preantral follicles in their ovaries which could respond to an *In Vitro* Activation (IVA) therapy to develop into larger follicles and to generate mature eggs for pregnancy.^6,7^ Ultrasound and MRI are commonly used in clinics to diagnose follicle growth and ovarian tumors.^38,39^ For follicles smaller than the secondary follicle (diameter < 100 μm), which are the majority of follicles, both ultrasound and MRI are ineffective. Because the prevailing transvaginal ultrasound approach^5^ does not allow imaging of preantral follicles, attempts have been made to use serum Anti-Mullerian Hormone (AMH) levels to predict the presence of preantral follicles.^23^ But in clinics, some patients with undetectable AMH levels could still respond to the IVA treatment due to the existence of residual preantral follicles.^24^

We used ultrasound and magnetic resonance imaging (MRI) approaches (ESI Fig. S4A and B†) to perform *in vivo* imaging of a mouse ovary but only obtained a low quality outline of large preovulatory follicles. We also used non-targeting single-walled carbon nanotubes (SWNCTs)^15^ to achieve live NIR-II imaging of the overall vascularity *in vivo* (Fig. S4C†). Although blood vessels surrounding ovarian follicles were detected, the signals were not specific and were found throughout all vasculature. The present use of specific FSH-CH allows for noninvasive imaging of FSH receptors in both antral and preantral follicles *in vivo*, thus providing the most sensitive and specific approach to detect follicles. Further improvement of the present FSH-CH approach and design of a portable transvaginal NIR-II probe could allow live imaging of preantral follicles in infertile female patients to be used as a diagnostic tool. For male infertile patients, NIR-II imaging of testes seminiferous tubules could also allow for better diagnosis of Sertoli cell functions in patients with oligospermia due to Sertoli cell defects.^25^

An earlier study demonstrated that osteoclasts and their precursors possess Gi2α-coupled FSH receptors that activate MEK/Erk, NF-κB, and Akt to enhance osteoclast formation and promote bone loss.^9^ Due to the difficulties involved in studying osteoclasts embedded in the rigid bone structure and a lack of *in vivo* tools to perform live imaging of bones expressing FSH receptors, earlier studies relied upon the use of isolated osteoclasts and their precursors expressing extremely low levels of FSH receptors. In addition, these findings have been challenged by another group showing no FSH receptor expression in mouse cultured osteoclasts or bones.^10,11^ Consistent with the expression of functional FSH receptors in osteoclasts,^9^ our study using live animals provides direct evidence for the binding of the FSH-CH ligand to FSH receptors in vertebrae and other bones in both sexes. To our knowledge, this is the first *in vivo* molecular imaging of the FSH receptor in bones.

Menopause-associated diminishment of bone density and bone fracture is a major health issue in modern society.^26^ The present CH1055 fluorophore has a low cell toxicity and short half-life *in vivo*.^16^ After ruling out potential side effects of the present fluorophore, the present NIR-II approach could provide valuable tools for *in vivo* evaluation of osteoclast functions in bones under different clinical conditions. Although estrogen replacement is routinely used to minimize fracture risks, side effects of estrogen include coronary heart disease, stroke, thromboembolic events, and breast cancer.^27^ Because FSH blocking antibodies prevent bone loss by inhibiting bone resorption and stimulating bone synthesis,^28^ our demonstration of bone FSH receptors *in vivo* provides the basis for designing FSH receptor antagonists to prevent bone loss in postmenopausal women contraindicated for the estrogen replacement therapy. The FSH receptor has also been shown to affect stemness and proliferation in mesenchymal stem cells (MSC) associated with osteoclast prevalence in bone marrow.^29^ FSH receptors are also expressed by endothelial cells in the blood vessels of a wide range of tumors in patients.^30,31^ The present approach could provide new tools for monitoring tumor progression *in vivo*.^32^ There are only a few studies dealing with molecular imaging reagents with sensitivity to the FSH receptor,^33^ the others focus on FSH receptor expressing cancer cells/tumors and vasculature associated with tumor development and progression.

The FSH receptor belongs to a subgroup of G-protein-coupled, seven-transmembrane receptors,^34^ consisting of receptors for the luteinizing hormone (LH)^35^ and thyroid stimulating hormone (TSH).^36^ Based on the present approach, the conjugation of a NIR-II fluorophore to the paralogous LH or TSH in the future could allow imaging of specific target cells expressing LH receptors (theca, luteal, and mature granulosa cells in the ovary and Leydig cells in the testes) and TSH receptors (thyroid nodules). Evaluation of LH conjugated NIR-II fluorophores could allow better monitoring of Leydig cell tumors whereas TSH conjugated fluorophores allow the evaluation of malignant and benign neoplasms of the thyroid in patients.^37^ Because the present fluorophore CH1055 has minimal cell toxicity and is rapidly excreted from the body,^16^ further refinement of the present NIR-II fluorophore conjugation approach could open ways to perform *in vivo* live imaging of receptors for diverse peptide and protein hormones in their target tissues.

## Author contributions

A. J. H., H. D., Y. F., A. A. and Z. C. conceived and designed the study, supervised the project, and wrote the manuscript. Y. F., A. A., Y. W., S. H. R. and S. Z. designed the study, performed all of the experiments, and wrote the manuscript. X. H., H. C., Y. X., A. A. and S. Z. contributed to the synthesis of the compounds, and A. A., S. Z., Y. F., X. L., L. J., K. Y. and S. D. assisted in the optical imaging.
