# Supplementary material for: Live imaging of follicle stimulating hormone receptors in gonads and bones using near infrared II fluorophore
Source: Chem Sci. 2017 Mar 6;8(5):3703–11. doi: 10.1039/c6sc04897h (PMC5465568; doi:10.1039/c6sc04897h)
Supplement: Supplementary file 1 [file SC-008-C6SC04897H-s001.pdf]

Electronic Supplementary Material (ESI) for Chemical Science.
This journal is © The Royal Society of Chemistry 2017

# Supplemental materials

## Methods

### *Animals*

Female Balb/c mice at days 17 and 21 of age, together with adult females and males at 9 weeks of age were obtained from Charles River Laboratories (Wilmington, MA). Animals were housed in animal facilities at Stanford University under 12 h dark/light schedules with free access to food and water. Animal Ethics Committees approved experimental protocols.

### *Primary Culture of Ovarian Granulosa Cells and the U87MG and MDA-MB-468 cancer cell line*

Day 21 female mice were injected intraperitoneally with a single dose of eCG (equine gonadotropin, 5 IU). At 48 h later, ovaries were dissected and put in 37℃ collection medium (500 ml DMEM F-12 containing 100U/ml penicillin-streptomycin, 10% fetal bovine serum and 0.1% BSA). After rinsing ovaries for two times, the follicular wall was punctured gently with the oblique end of 0.5 ml Latex free syringe needles (gauge 28.5) to obtain granulosa cells in the form of a semitransparent milky cloud of cells. Cells were pooled and collected (1 ml/tube) by aspiration into a syringe or Pasteur pipette and transferred into a small 1.5 ml non-sticky Eppendorf tube. After centrifugation, supernatant was discarded and cells washed for two times. Cells were adjusted to a concentration of $1\times10^{6}$/200 µl, before culturing at 37C in a 5% CO2 incubator for *in vitro* experiments. The U87MG and MDA-MB-468 mammary cancer (MDA) cell lines were obtained from the American Type Tissue Culture Collection (Manassas, VA) and maintained in culture.

### Synthesis and Optical Characterization of the NIR-II fluorophore CH1055

Synthesis of a small-molecule organic NIR-II dye (CH1055, MW 0.97 kDa, Fig. 1A) was achieved with high yield using 4,4'-(phenylazanediyl) dibenzaldehyde (Sigma-Aldrich Chemical Co., St. Louis, MO). Key steps utilized to assemble the core structure of the target included a cross-Suzuki coupling reaction, iron reduction, and N-thionylaniline induced ring closure [37]. Four carboxylic acid groups were introduced into a donor-acceptor-donor (D-A-D) type fluorescent compound to impart aqueous solubility and to allow facile conjugation to targeting ligands. In order to further increase solubility, the carboxylic acid groups of CH1055 were PEGylated with 2 kDa PEG-$NH_2$ through EDC/NHS chemistry (Fig. 1A). Successful PEGylation was confirmed with MALDI-TOF-MS, demonstrating the 8.9 kDa conjugate to be the predominant species. A fluorescent emission spectrum was taken with the 808 nm excitation laser used for *in vivo* imaging which aligned well the photoluminescence excitation/emission mapping. Superior photostability of CH1055-PEG was observed by exposing the conjugate in water, PBS, and serum to a continuous 808 nm laser at a power density of 0.33 W/$cm^2$. The quantum yield of CH1055-PEG was on par with the majority of NIR-II nanomaterials including carbon nanotubes and thus suitable for *in vivo* imaging [38-40].

***Conjugation of FSH to the NIR-II dye***

Conjugation between recombinant human FSH (Sigma-Aldrich Chemical Co.; St. Louis, MO) and the NIR-II dye CH1055 (M.W. 0.97 kDa) was based on the 1-ethyl-3-(3-dimethylaminopropyl) carbodiimide hydrochloride (EDC) method as previous described [18]. EDC was used to react with the carboxyl groups on the surface of the CH1055 dye, forming an unstable reactive O-acylisourea intermediate. Subsequently, the amine groups of proteins were covalently conjugated to the reactive O-acylisourea modified CH1055 to produce FSH-CH. For a typical reaction, 100 µL FSH (3.52 µM) was added in 83.33 µL PBS; then 43.33 µL CH1055 (602 µM in DMSO) was added drop by drop. After 10 min., 2.3 µL of EDC (10 mM) was added and the reaction mixture was stirred for 12 h at 4 °C. To isolate FSH-CH, a centrifuge filter with

a cutoff of 30 kDa (Amicon) was used to remove un-conjugated CH1055 that was not water-soluble. The final volume of FSH-CH for injection was 150 μL.

***In vitro testing of FSH-CH***

Lysate was extracted from granulosa and U87MG cells, respectively, according the standard protocol (CelLytic M, Sigma, St. Louis, MO). The concentration of cell lysate was determined by BCA protein assay kit before printing cell lysates (2 mg/mL) on plasmonic NIR fluorescence-enhancing Au slides. After blocking the slide by using 50 mg/mL bovine serum albumin for 0.5 h, 100 μL purified FSH-CH (35.2 nM) was added on the slide and incubated for 1h. The slides were then washed by using phosphate buffered saline with Tween-20 for five times and the dried slides were scanned using NIR fluorescent camera with 808 nm laser excitation and 1100 nm long pass filter.

***Live NIR-II imaging***

Mice were shaved using Nair hair removal cream and anesthetized using Isofluane (Piramal Group) before placing in a stage with a venous catheter for injection of imaging agents. All NIR-II images were collected on a 320 × 256 pixel two-dimensional InGaAs array (Princeton Instruments). The excitation laser was an 808 nm laser diode at a power density of ~0.3 $W/cm^2$. Emission was typically collected with a 1,100 nm LP filter. A lens set was used for obtaining tunable magnifications, ranging from 1x (whole body) to 12.5x (high magnification) by changing the relative position of two NIR achromats (200 mm and 75 mm, Thorlabs). A variable exposure time was used for the InGaAs camera (320 × 256 pixel) to capture images in the NIR-II window. Images were processed with Matlab.

*MALDI-TOF-MS*

Matrix assisted laser desorption/ionization time of flight mass spectrometry (MALDI-TOF-MS) by Stanford Protein and Nucleic Acid Biotechnology Facility, Stanford University. Analytical or preparative high performance liquid chromatography (HPLC) was performed on a DIONEX ultimate 3000 instrument with PDA detection (column: PrincetonSPHER-300 $C_{18,}$ 5μ, 250 mm ×4.6 mm or 10.0 mm; mobile phase: water/acetonitrile with 0.1 % TFA).

# References


1 G. Qian, B. Dai, M. Luo, D. Yu, J. Zhan, Z. Zhang, D. Ma and Z. Y. Wang, *Chemistry of Materials*, 2008, **20**, 6208-6216.

2 S. Diao, G. Hong, J. T. Robinson, L. Jiao, A. L. Antaris, J. Z. Wu, C. L. Choi and H. Dai, *J Am Chem Soc*, 2012, **134**, 16971-16974.

3 G. Hong, Y. Zou, A. L. Antaris, S. Diao, D. Wu, K. Cheng, X. Zhang, C. Chen, B. Liu and Y. He, *Nature Communications*, 2014, **5**.

4 S.-Y. Ju, W. P. Kopcha and F. Papadimitrakopoulos, *Science*, 2009, **323**, 1319-1323.

5 A. L. Antaris, H. Chen, K. Cheng, Y. Sun, G. Hong, C. Qu, S. Diao, Z. Deng, X. Hu and B. Zhang, *Nat. Mater.*, 2016, **15**, 235-242.

**Supplemental figures**

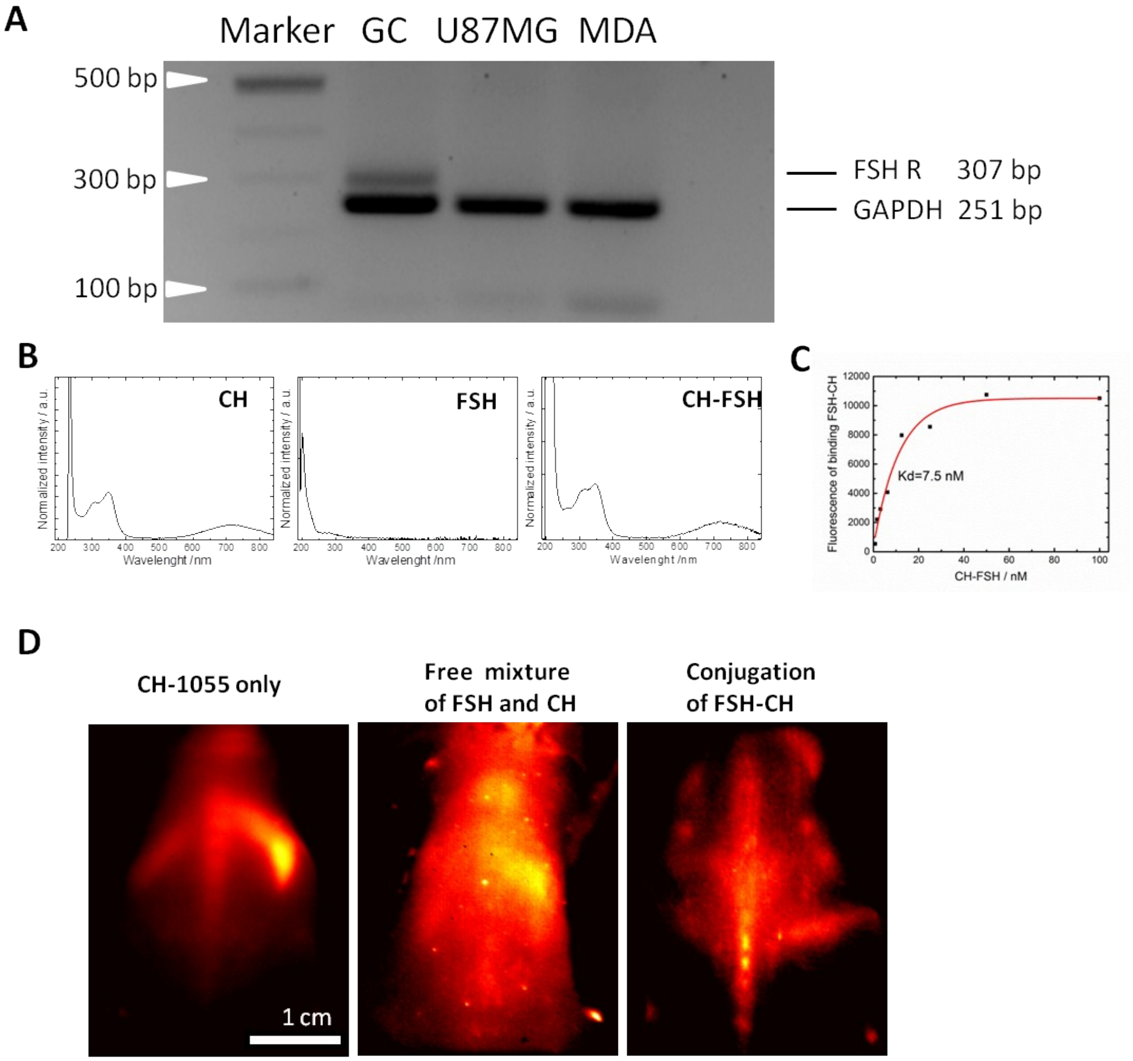


**Fig. S1. The specific and affinity analysis of FSH-CH.** (A) FSH receptor mRNA was detected in ovarian granulosa cells (GC), while not in those of U87MG and MDA cell lines. Primers for FSHR (301 bp): F:5- GCTGTGCCTTTGCAAACTGGAG-3; R: ATGGCCAGGATGCTGATAAACC. Primers for GAPDH (250 bp): F: CCATGTTCGTCATGGGTGTGAACCA; R: GCCAGTAGAGGCAGGGATGATGTTC.

(B) The UV-vis absorption spectra of CH free FSH and CH-FSH conjugation were measured respectively. The absorption peak of FSH is ca. 202 nm with tail to 300 nm;

and the absorption peaks of CH are 233, 350 and 720 nm. After forming the conjugation, there is new broad peak from 200-235 nm. (C) The scatchard plot was obtained by testing fluorescence intensity of cultured GC cells with gradient concentrations of CH-FSH, and it showed that the Kd value is 7.5 nM. (D) *In vivo* experiments using CH  only, mixture of FSH and CH as well as the conjugation FSH-CH  showed that injection with FSH-CH had specific signals in targeted tissues.

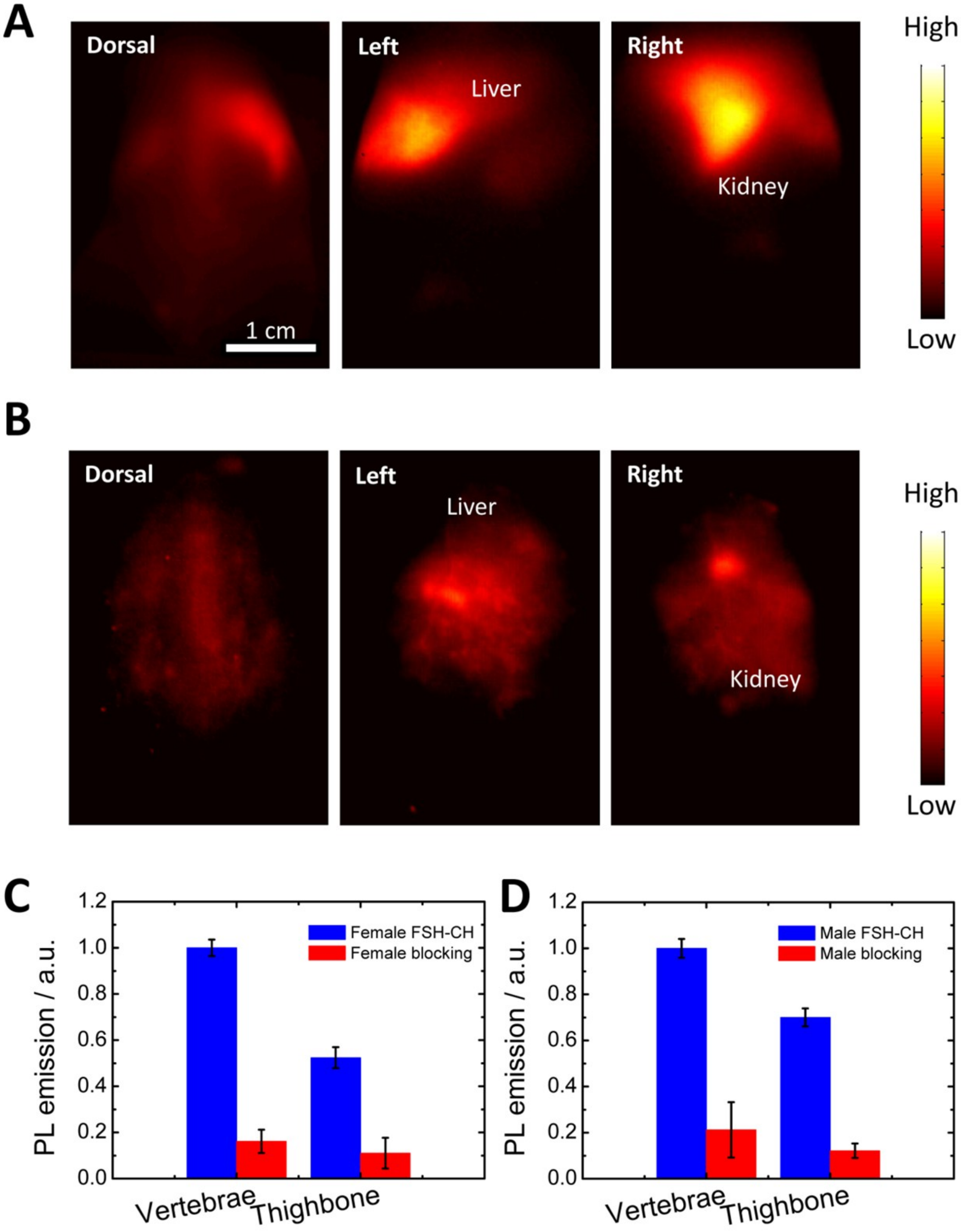


**Fig. S2. Specificity of follicle stimulating hormone-fluorophore CH1055 (FSH-CH) signals in bones.** Specific NIR-II signals in both (A) female and (B) male mice were blocked when FSH-CH (12.5 μg) was injected together with 20-fold excess of non-

conjugated FSH. Non-specific signals were found in liver and kidney only. Quantitation of NIR-II signals in individual groups in two types of bones in both (C) female and (D) male mice. Errors bars indicate the standard deviation of each group. PL, photo luminescence.

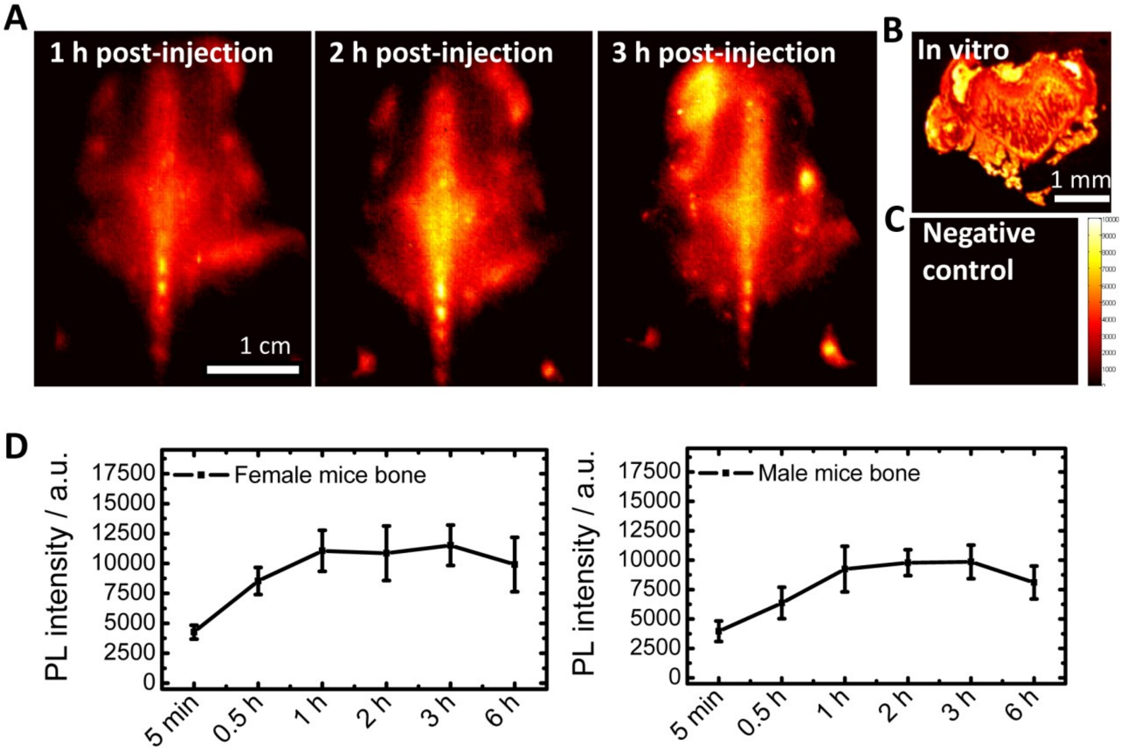


**Fig. S3. NIR-II imaging for pharmacokinetics of bones with follicle stimulating hormone-fluorophore CH1055 (FSH-CH) (12.5 μg)**. (A) FSH-CH (12.5 μg) was injected into the tail vein of an adult female mouse before NIR-II imaging at different post-injection times. Bone signals begun to show up around 1 h after injection, then showing an increase in the subsequent 2 hours. (B) Bone section with FSH-CH using NIR-II microscope (×15 times, 12.5 μg) *in vitro* at 24 h after injection. (C) Bones section without FSH-CH were regarded as the negative control. (D) Quantitation of fluorescence intensity in the bone at different time point in adult males and females (n=3).

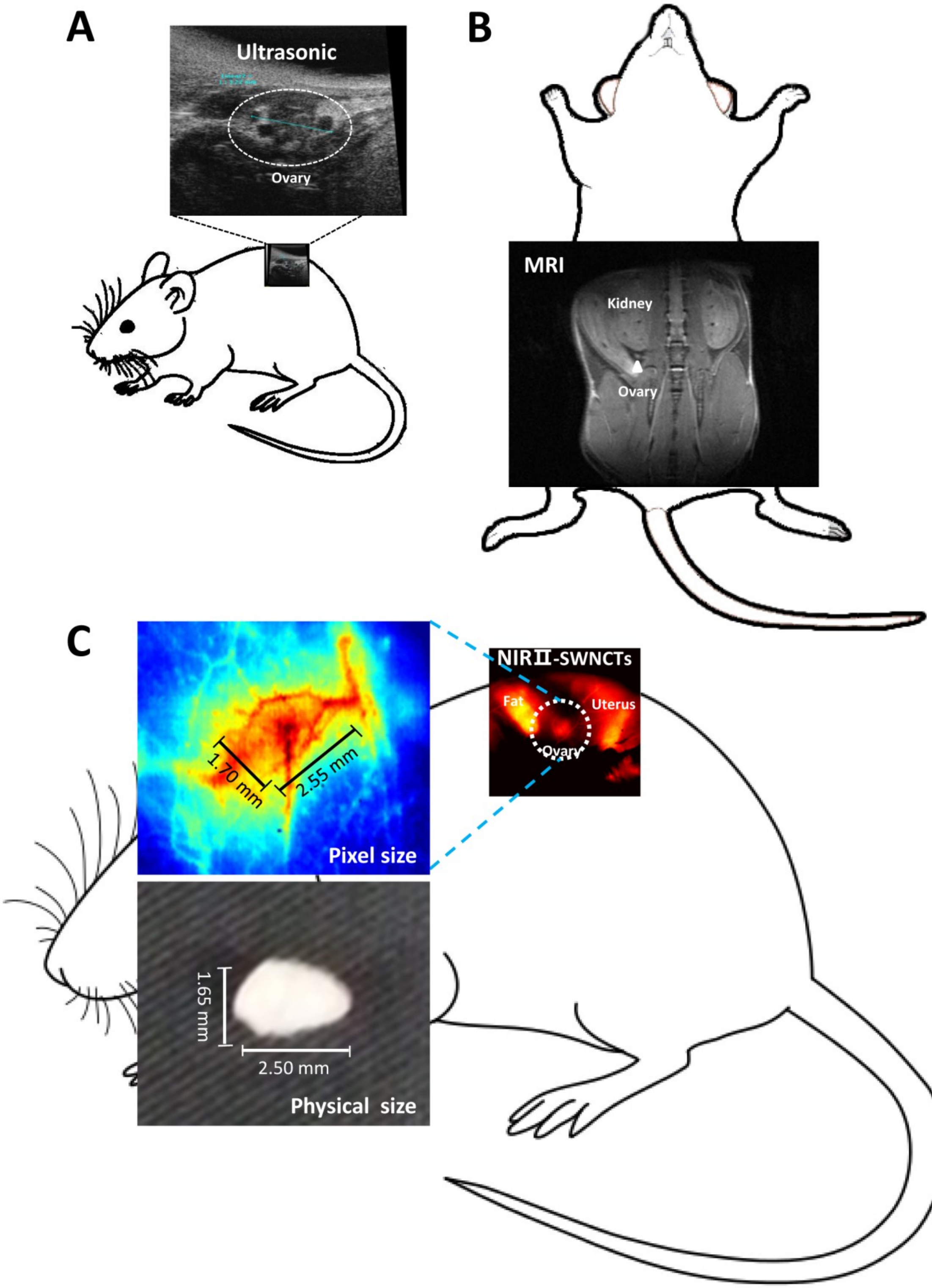


**Fig. S4. Three methods of mouse ovary imaging.** (A) Ultrasound image of the ovary in an adult female mouse showing blurred signals of large follicles. (B) Magnetic resonance

imaging (MRI) of an adult female mouse showing the outline of an ovary without revealing follicle structures. (C) NIR-II imaging of the ovary using single-walled carbon nanotubes (SWNCTs) to reveal vascularity. Left panel: Whole body view. Note signals in the ovary and spleen. Right upper panel: higher magnification showing NIR-II signals in blood vessels inside the ovary. Right lower panel: actual physical size of the ovary.

**Supplemental video**

Video S1. **NIR-II imaging of ovarian follicles using follicle stimulating hormone-fluorophore CH1055 (FSH-CH) in adult female mice: time-course and specificity.** FSH-CH (12.5 μg/200 μl) was injected into the tail vein of an adult female mouse under the NIR-II camera at different post-injection times. Note rapid accumulation of signals in the blood vessels and kidney at 60 s, followed a decline. Ovarian signals begun to show up at 120 s and peaked at 2-6 h after injection, showing a sustained retention for up to 24 h. Side view images are shown to focus on one ovary.
